# Genetic characterization of the marmot gut virome in high-altitude Qinghai Province and identification of novel viruses with zoonotic potential

**DOI:** 10.1128/msphere.00297-25

**Published:** 2025-07-31

**Authors:** Haisheng Wu, Xiaojie Jiang, Yuan Xi, Songyi Ning, Hailian Wu, Wenyuan Xin, Wenxuan Peng, Shengjun Wang, Wen Zhang

**Affiliations:** 1 Department of Microbiology, School of Medicine, Jiangsu University, Zhenjiang, Jiangsu, China; 2 Qinghai Institute for Endemic Disease Prevention and Control 648562, Xining, China; 3 Department of Clinical Laboratory, Wuxi Blood Center, Wuxi, Jiangsu, China; Instituto de Biotecnologia/UNAM, Cuernavaca, Morelos, Mexico

**Keywords:** marmot, viruses, phylogenetic, viral metagenomics

## Abstract

The high-altitude ecosystems of Qinghai Province, China, harbor unique viral communities shaped by extreme environmental conditions and host adaptations. This study presents a comprehensive genetic characterization of the marmot gut virome, revealing novel viral strains with zoonotic potential. Using viral metagenomics, we analyzed intestinal contents from 70 marmots collected from Chengduo and Maqin counties. Sequencing on the Illumina NovaSeq 6000 platform identified 19 viral genomes belonging to four major families: *Adenoviridae*, *Astroviridae*, *Parvoviridae*, and *Picornaviridae*, along with four novel circular Rep-encoding single-stranded DNA (CRESS DNA) viruses. Phylogenetic analyses demonstrated close relationships between marmot-derived strains and viruses from humans, bats, and other mammals, highlighting potential cross-species transmission risks. Notably, bat-associated adenoviruses showed closer phylogenetic proximity to human strains, while novel parvoviruses formed a distinct clade within the Dependoparvovirus genus. The discovery of a novel astrovirus with low sequence similarity to known genera underscores the need for taxonomic reclassification. Additionally, a novel picornavirus related to Sapelovirus and four divergent CRESS DNA viruses were identified, expanding our understanding of viral diversity in high-altitude rodents. These findings emphasize the role of marmots as viral reservoirs and highlight the importance of high-altitude ecosystems as hotspots for zoonotic pathogen emergence. This study provides critical insights into viral evolution, host adaptation, and zoonotic risks, advocating for integrated surveillance strategies to mitigate future spillover events.

**IMPORTANCE:**

Viruses are the most abundant and diverse biological entities on Earth, yet their presence in wildlife from extreme environments remains poorly understood. High-altitude ecosystems, shaped by harsh conditions like intense UV radiation and low oxygen levels, create unique settings for virus evolution. This study is the first to comprehensively profile the gut virome of marmots in Qinghai Province, uncovering novel viral strains and highlighting how extreme environments drive viral diversity. Marmots, as key species in these regions, can act as bridges for virus transmission among wildlife, livestock, and humans, posing zoonotic risks. Understanding these viral communities is essential for predicting and preventing future outbreaks. Our findings emphasize the urgent need for integrated, One Health-based surveillance strategies to safeguard both public health and biodiversity in fragile high-altitude ecosystems.

## INTRODUCTION

Viruses are the most abundant biological entities on Earth, with an estimated global population of 10^31^ particles, representing an unparalleled genetic diversity. Despite this vast presence, methodological constraints have meant that over 99% of viral taxa remain uncharacterized (1, 2). Although significant strides have been made using viral metagenomics and other advanced techniques to map global viral diversity, major knowledge gaps persist—especially concerning the viromes hosted by wildlife in extreme environments.

High-altitude ecosystems, typically defined as regions exceeding 2,500 m, present a suite of environmental stressors including intense ultraviolet radiation, chronic hypoxia, and marked temperature fluctuations (3). These conditions impose unique evolutionary pressures on resident viral communities, fostering adaptations that are distinct from those observed in lower-altitude or more temperate habitats (4). Such ecosystems serve as natural laboratories for investigating virus–host coevolution, where the interplay of extreme environmental factors and host biology can lead to unexpected patterns of viral persistence and diversification (5).

Among the species inhabiting these challenging environments, marmots (Marmota spp.) are of particular scientific interest. These rodents are not only ecologically significant in Asian alpine grasslands, where they function as keystone species, but also possess biological traits—such as prolonged hibernation cycles and dense population clustering—that may favor viral maintenance and evolution (6). Marmots’ burrowing behaviors significantly alter their ecosystems by aerating soil and redistributing microbial communities, while their fecal matter contributes to the contamination of water sources used by grazing yaks and local pastoral communities. This ecological interconnectedness creates a complex transmission network through which viruses may circulate among wildlife, domesticated animals, and humans (7). A striking example of the public health risks associated with such zoonotic interfaces occurred in Tibet in 2024, when an outbreak of bubonic plague, linked to dry otter-associated transmission, resulted in fatalities among local herders (8). This incident underscores the potential for high-altitude regions to serve as hotspots for the emergence and spillover of zoonotic pathogens, especially in areas where traditional pastoral practices and limited healthcare infrastructure amplify the risks (9). It also highlights the critical need for enhanced surveillance and early-warning systems capable of detecting emerging threats before they can escalate into widespread outbreaks.

Despite these concerns, the role of the marmot gut as a viral reservoir remains inadequately explored. A marmot-linked plague occurrence was documented in Tibet in 2024, leading to fatalities within the local herder community (10). In high-altitude environments, where hypoxic conditions can alter the composition of the host gut microbiota, there is a strong possibility that changes in phage–bacterial interaction networks may drive viral recombination events and further diversify the virome (11). Moreover, the extreme environmental pressures characteristic of these regions may select for viral particles with enhanced stability, thereby increasing the probability of transmission via contaminated water or aerosolized particles (12).

Given these complex interactions, a systematic analysis of the genetic composition of the marmot gut virome is imperative. Such research would not only provide insights into the mechanisms underlying viral evolution in extreme conditions but also furnish critical data for developing early-warning systems aimed at preventing zoonotic outbreaks. The advent of unbiased next-generation sequencing and other metagenomic approaches has now made it feasible to comprehensively profile viral communities in complex samples, including those viruses that escape detection by conventional culture-based methods (13). Integrating these cutting-edge techniques within a One Health framework will be essential for establishing a robust surveillance system that addresses infectious disease risks across the wildlife–livestock–human continuum (14).

In light of the increasing frequency of zoonotic events and their severe socio-economic implications, advancing our understanding of the marmot gut virome is a research priority. Unraveling the diversity and evolutionary trajectories of these viral communities will not only expand our fundamental knowledge of virus–host dynamics in high-altitude ecosystems but also provide the empirical basis for predicting and mitigating the impact of future zoonotic transmissions (15).

## MATERIALS AND METHODS

### Collection of Marmota samples

To perform a comprehensive viral metagenomic analysis, we captured 70 adult wild Himalayan marmots (*Marmota himalayana*) near human settlements in Qinghai Province, China, in 2023. The specimens included 50 individuals from Chengduo County, Yushu Prefecture (approximate elevation: 4,200 m; mean annual temperature: −1.5°C), and 20 individuals from Maqin County, Golog Prefecture (elevation: ~3,800 m; mean annual temperature: −2.0°C). Both locations lie on the eastern Tibetan Plateau and are characterized by high-altitude alpine environments with cold climates and limited vegetation cover. All field operations and subsequent laboratory dissections were performed by personnel wearing Class C personal protective equipment in accordance with biosafety protocols.

Intestinal contents from each marmot were aseptically collected in 50 mL sterile centrifuge tubes and preserved at −80°C for a minimum stabilization period of 7 days prior to processing. The experimental protocols, including sample collection procedures, were formally approved by the Institutional Animal Care and Use Committees of Jiangsu University and Qinghai Institute for Endemic Disease Control and Prevention. All specimens were immediately transferred to −80°C ultra-low temperature freezers following collection to ensure optimal biomolecular preservation.

Notably, the entire study strictly adhered to the ethical guidelines established by both approving institutions, with particular emphasis on humane animal handling practices and environmental impact mitigation. Sample pretreatment procedures were conducted under biosafety level two containment conditions at Qinghai Institute for Endemic Disease Control and Prevention, incorporating redundant biocontainment measures including negative-pressure laboratories and high efficiency particulate air (HEPA) filtered ventilation systems.

### Handling of intestinal content samples

Each marmot’s intestinal content sample was processed as an independent library, establishing a total of 70 individual libraries. Intestinal contents were homogenized using a cryogenic grinder, resuspended in 1 mL of Dulbecco’s phosphate-buffered saline, and rapidly frozen at −80°C (16). After three freeze-thaw cycles with mechanical grinding, samples were centrifuged (15,000 × *g*, 10 min) to collect the supernatants. The supernatant was filtered through 0.45 µm membranes (Millipore) to remove eukaryotic and bacterial cellular debris (17, 18).

A 200 µL aliquot from each sample pool was treated with a DNase/RNase enzyme mixture (37°C, 60 min) for complete nucleic acid digestion (19). Residual nucleic acids were extracted using the QIAamp Viral RNA Mini Kit (Qiagen) following the manufacturer’s instructions. As RNA constituted the primary nucleic acid component, reverse transcription was performed using SuperScript IV Reverse Transcriptase with random hexamer primers (20).

Double-stranded DNA (dsDNA) synthesis was achieved through Klenow fragment polymerase (New England Biolabs) treatment, converting both cDNA and single-stranded DNA viral genomes into dsDNA templates. Libraries were prepared from the dsDNA products using the Illumina Nextera XT DNA Library Preparation Kit, generating 70 individual libraries for subsequent sequencing analysis.

### Genomic sequencing and bioinformatics analysis

Sequencing was conducted on the Illumina NovaSeq 6000 platform using 250 bp paired-end dual-indexed libraries, with subsequent demultiplexing of 70 samples via Illumina’s proprietary barcode system. Raw reads underwent host sequence removal through Bowtie 2 v2.3.4.1 alignment against bacterial/fungal genomes from GenBank, followed by quality control using Phred v1.0.0 (Q10 trimming threshold) and position-specific duplicate elimination (identical sequences at positions 5-55) (21). Residual adapters were trimmed with NCBI’s VecScreen using default parameters. Processed reads were assembled through parallel strategies: ENSEMBLE v1.0.0 for initial contig generation and Geneious Prime v2019.0 for viral sequence refinement (22, 23). Viral identification involved DIAMOND BLASTx v2.0.15 screening against a custom non-viral non-redundant (NVNR) database (e-value ≤ 10^−5^), with subsequent validation against NCBI viral proteome/nr database entries (16). Sequences demonstrating superior alignment to NVNR were excluded as non-viral. Unclassified contigs underwent HMMER3 v3.3.2 analysis against the vFam database to detect distant viral homologs (24). Open reading frame prediction and functional annotation integrated BLASTx results with conserved domain analysis in Geneious Prime. Taxonomic classification was finalized through MEGAN v6.21.16’s LCA algorithm, utilizing DIAMOND-generated BLASTx outputs converted from daa to rma6 format for comprehensive viral profiling.

### Integrated virome analysis

Statistical characterization of viral communities across 197 libraries was performed through comparative composition analysis in MEGAN v6.21.16, complemented by R v4.2.1-based visualizations (pheatmap and vegan packages) for structural profiling and abundance mapping (25). Temporal viral dynamics were graphically represented using ggplot2 in R, with statistical significance thresholded at *P* < 0.05. For phylogenetic reconstruction, viral open reading frames (ORFs) predicted through Geneious Prime v2019.0 and NCBI database alignments were compiled in MEGA v11.0.13 (26). Bayesian inference was conducted in MrBayes v3.2.7 under mixed amino acid substitution models (“prset aamodelpr=mixed”) across 10 million Markov chain Monte Carlo generations, terminating when the average standard deviation of split frequencies reached <0.01 (27). Final phylogenetic trees integrating top BLASTx matches from NCBI GenBank and representative family sequences were annotated and visualized through FigTree v1.4.4 and Adobe Illustrator 2020 v26.0.1, ensuring both analytical rigor and graphical precision (25).

### Quality control

To minimize nucleic acid contamination risks, autoclaved double-distilled water (ddH_2_O) served as process-matched negative controls throughout experimental workflows. All sample-handling procedures adhered to stringent biosafety protocols to prevent cross-contamination and nucleic acid degradation. Critical consumables demonstrated DNase/RNase-free certification, while nucleic acid dissolution utilized diethyl pyrocarbonate-treated water supplemented with RNase inhibitors to ensure enzymatic inactivation. Parallel processing of controls confirmed the absence of exogenous nucleic acid carryover in downstream analyses.

## RESULTS

### Overview of the intestinal virome

To investigate the intestinal virome of marmots in high-altitude regions, we collected intestinal contents from 70 marmots captured in Chengduo County, Yushu Prefecture (*n* = 50) and Maqin County, Golog Prefecture (*n* = 20), Qinghai Province, China. A total of 70 libraries were constructed from these samples and subjected to viral metagenomic sequencing using the Illumina NovaSeq 6000 platform.

**Fig 1:**
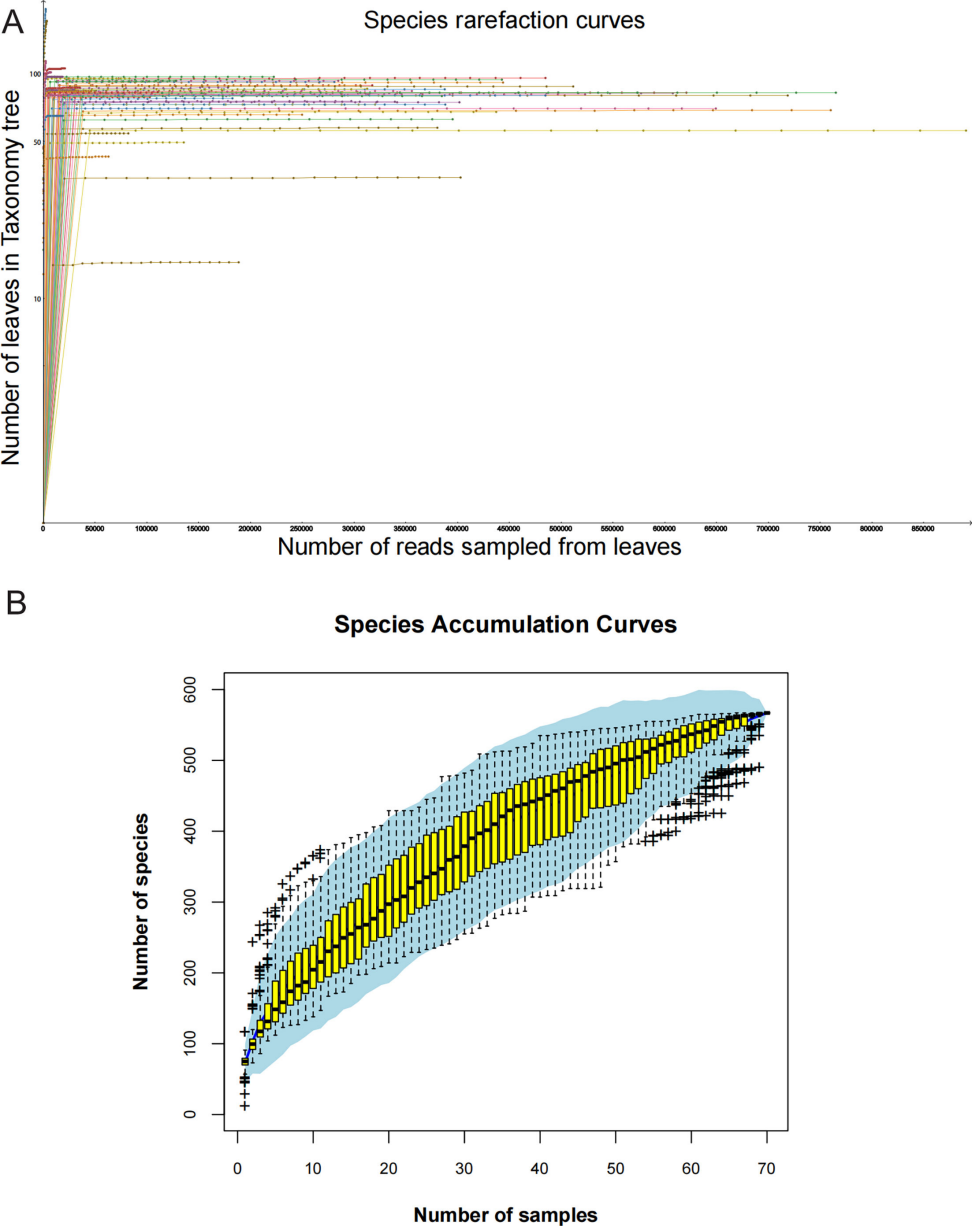
The diversity of viral species in the 70 libraries. (**A**)The resulting species rarefaction curves were plotted after log-scale transformation of the raw data in MEGAN v6.21.16 software. (**B**) Accumulation curve of viral species in canine metagenomes. Individual box plots in the plots correspond to the richness values of the samples, with light blue areas representing 95% confidence intervals.

Species rarefaction and accumulation curves demonstrated that viral species richness reached saturation in the 70 libraries, indicating sufficient sequencing depth to capture existing viral diversity with minimal likelihood of discovering additional species through further sequencing (Fig. 1A). The smoothing trend of accumulation curves with increasing sample size confirmed the representativeness of our sample collection for this study (Fig. 1B). Over 500 distinct viral species were identified across all libraries.

Subsequent characterization identified 19 viral genome sequences belonging to four major viral families: *Adenoviridae* (*n* = 4), *Astroviridae* (*n* = 1), *Parvoviridae* (*n* = 13), and *Picornaviridae* (*n* = 1), along with four circular Rep-encoding single-stranded DNA (CRESS DNA) viruses.

### Diversity analysis of virus communities

**Fig 2:**
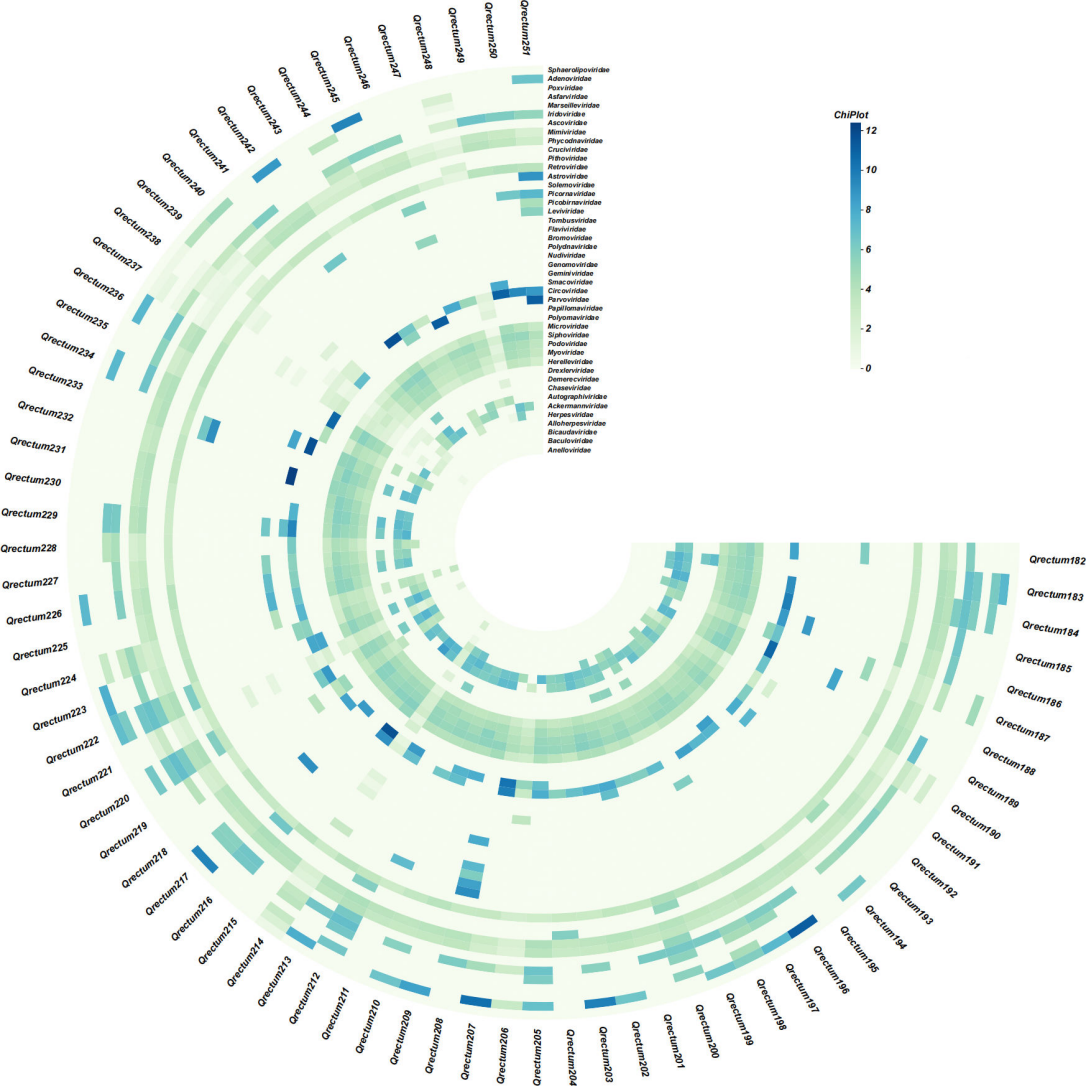
Heatmap visualization of gut virome composition. Viral family distributions were systematically analyzed through a heatmap generated using hierarchical clustering. Taxonomic classifications were color-coded according to the standardized legend, with chromatic intensity corresponding to relative abundance levels.

**Fig 3:**
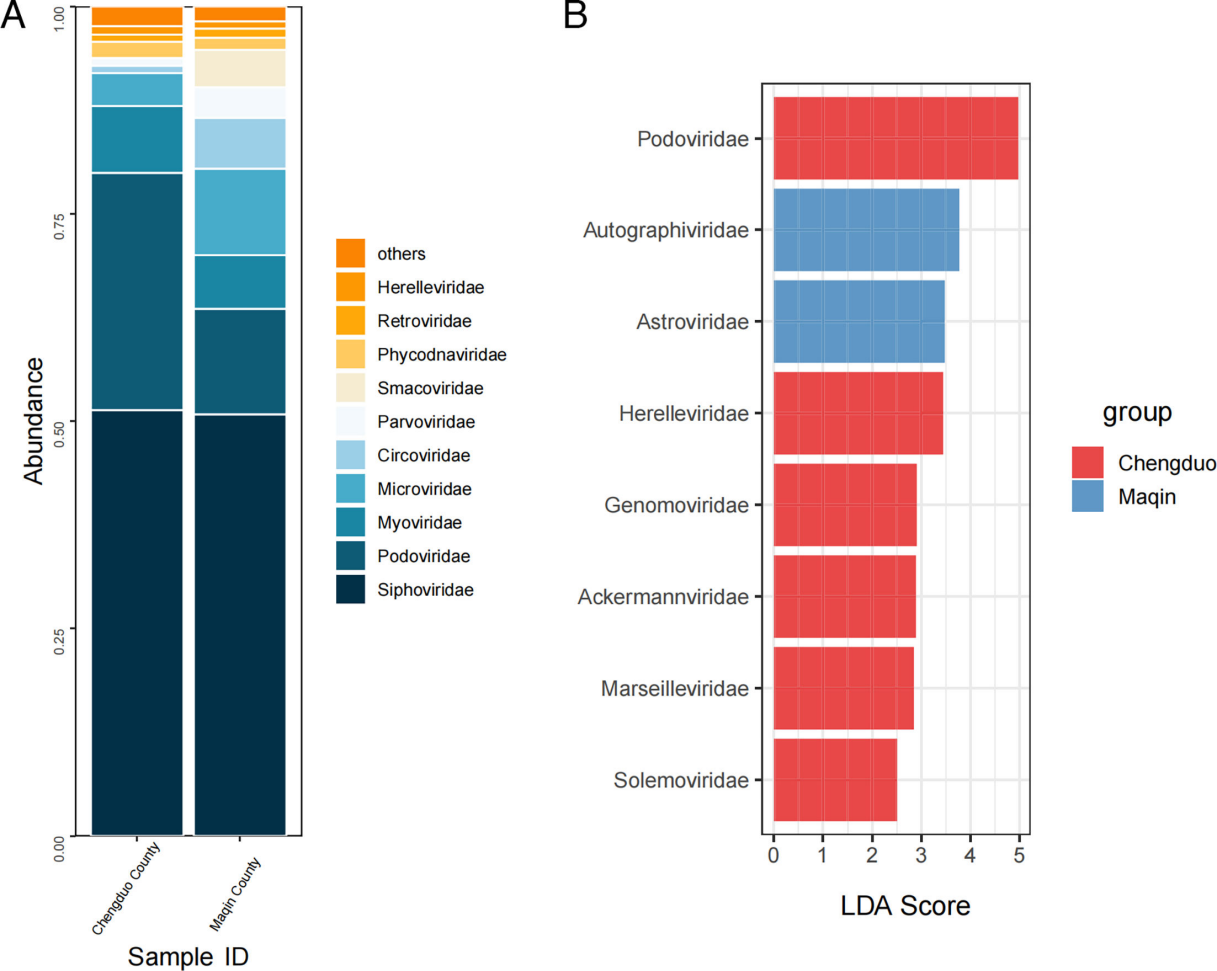
(**A**) Geographical stratification of viral taxonomic profiles. Comparative analysis of virome composition across geographical regions was performed through a stacked bar chart, depicting taxonomic composition and relative abundance of viral families. (**B**) Linear discriminant analysis effect size (LEfSe) of differential taxa between groups. The bar plot shows taxa with significantly different relative abundances between the compared groups, identified by LEfSe. Only taxa with an LDA score >2.0 and *P* value <0.05 are displayed. The length of each bar represents the effect size (LDA score), indicating the magnitude of difference.

Heatmap analysis of viral composition variations revealed 44 distinct viral families at the taxonomic level, with data visualization enhanced through logarithmic transformation (Fig. 2). A comparative analysis of viral taxonomic profiles between two geographical regions (Chengduo County and Maqin County), as illustrated in Fig. 3, revealed significant regional disparities in viral family abundance. Both regions exhibited comparably high abundance levels of *Siphoviridae* with minimal variation, indicating limited geographical influence on this viral family and demonstrating its environmental stability across distinct ecosystems (Fig. 3A). Furthermore, marked differences in relative abundance were observed when comparing viral families between the two counties. Notably, *Parvoviridae* demonstrated higher abundance in Chengduo County, whereas *Myoviridae* and *Microviridae* were more prevalent in Maqin County. Podoviridae exhibited the highest linear discriminant analysis (LDA) score, indicating that it plays a major role in distinguishing between the Chengduo and Maqin groups and contributes most substantially to the observed group differences (Fig. 3B). These observed divergences in viral family distribution suggest that geographical determinants—including local climatic conditions, ecosystem diversity, and host-pathogen interactions—play pivotal roles in shaping viral community structures.

**Fig 4:**
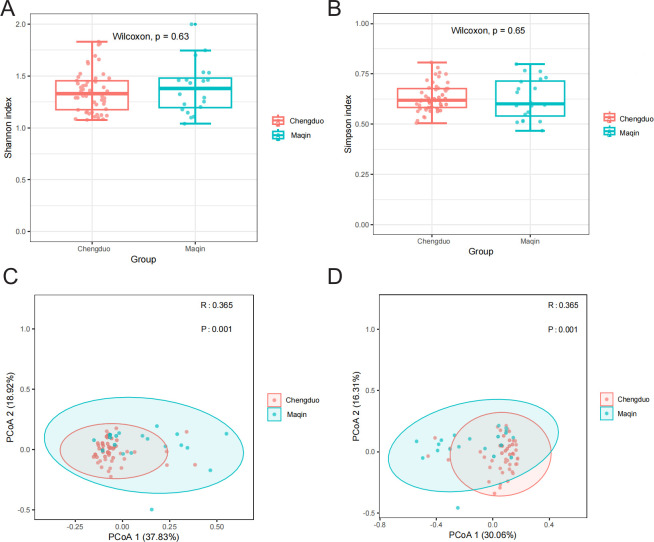
α-Diversity and β-diversity of gut virome communities. Prior to comparing viral α-diversity and β-diversity, viral sequences were normalized using MEGAN for subsequent analyses. α-Diversity was quantified at the family level using Shannon in panel A and Simpson indices in panel B as measurement parameters. Wilcoxon test was employed to calculate *P* values, with statistical significance determined when *P* < 0.05. To ensure accurate estimation of β-diversity, both Bray-Curtis and Jaccard metrics were adopted. The Bray-Curtis index was specifically utilized for β-diversity assessment in panel C, whereas the Jaccard index was applied for β-diversity analysis in panel D. Statistical significance was established when *P* < 0.05, and an *R* value >0 indicated intergroup dissimilarities.

Comparative analysis of α-diversity and β-diversity between Chengduo County and Maqin County revealed distinct ecological patterns (Fig. 4). The non-significant α-diversity indices (Fig. 4A and B) indicate comparable viral community characteristics in terms of species richness (total number of taxa) and evenness (equitability of species distribution) between the two regions. In contrast, significant β-diversity divergence (Fig. 4C and D) demonstrates substantial dissimilarities in overall viral community structure, manifested through differential species composition (presence/absence of specific taxa) and relative abundance patterns (proportional representation of constituent species). These structural variations in viral assemblages likely reflect environmental drivers shaping community organization across geographical locations.

### Phylogenetic analysis of *Adenoviridae*

Members of the family *Adenoviridae* possess linear double-stranded DNA genomes whose replication depends on virus-encoded DNA polymerase (28). This enzyme exhibits high conservation across adenovirus species, with minimal structural and functional variation, making it an ideal genetic marker for evolutionary studies within this viral family. Furthermore, the central role of DNA polymerase in the viral life cycle establishes it as a critical target for investigating replication mechanisms (29). Given its evolutionary conservation, functional indispensability, and extensive sequence data availability, the adenoviral DNA polymerase represents an optimal molecular marker for phylogenetic tree construction.

**Fig 5:**
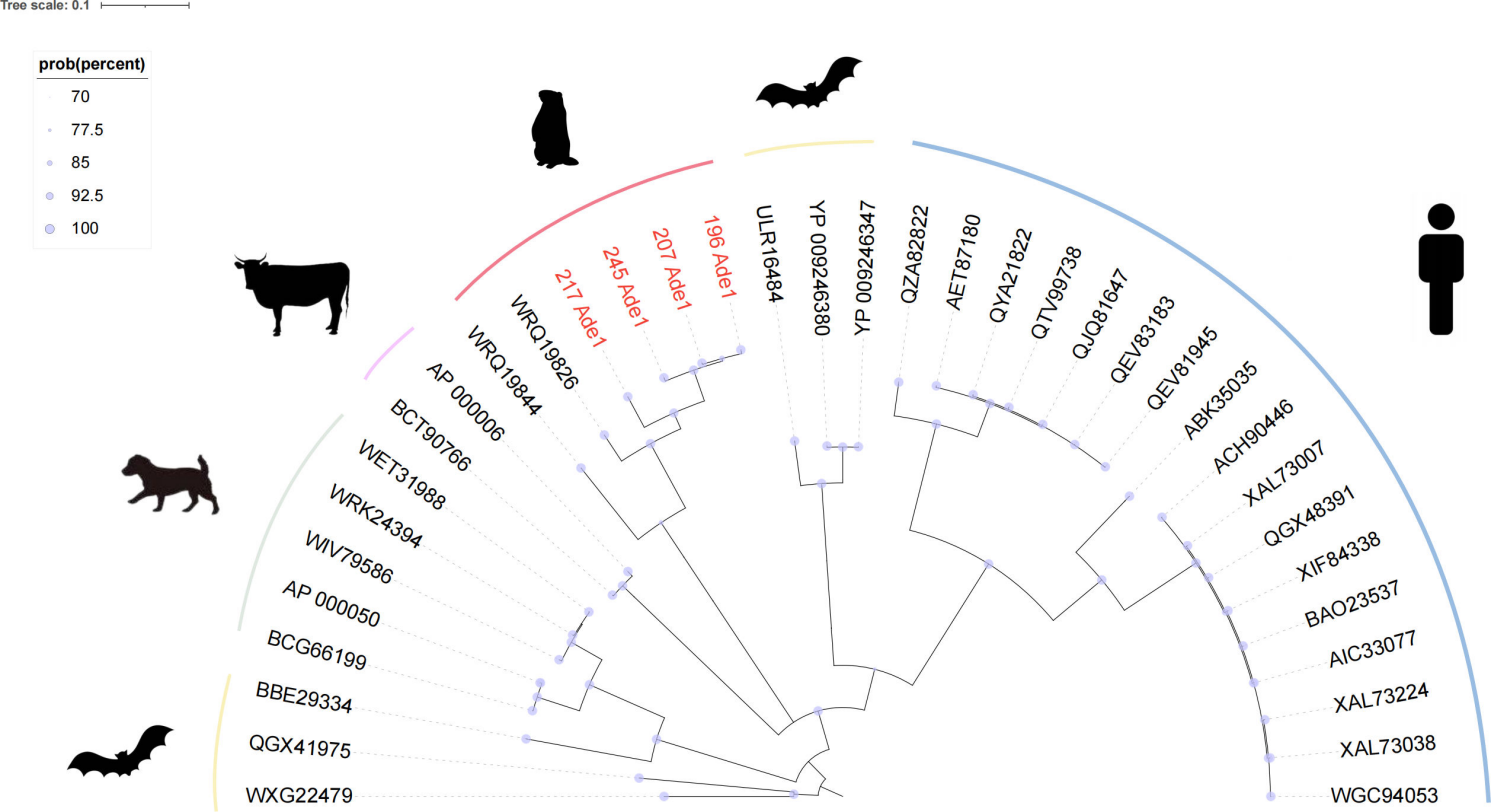
Phylogenetic analysis of *Adenoviridae*. Bayesian inference phylogenetic tree was constructed based on amino acid sequences of DNA polymerase from representative *Adenoviridae* viruses. Sequences obtained in this study are highlighted in red. The scale bar indicates the number of amino acid substitutions per site.

The phylogenetic tree (Fig. 5) clearly demonstrates that the red-marked nodes represent novel virus strains identified in this study, exhibiting well-defined phylogenetic relationships with known viruses. All reference viruses belong to the genus Mastadenovirus within the family *Adenoviridae*. Notably, the known viral sequences WRQ19826 and WRQ19844, collected from marmots in Xinjiang, China (2016), clustered with the four novel strains identified in this study, indicating a broad geographical spread of *Adenoviridae*. Interestingly, strains 196 Ade1 and 207 Ade1, collected from marmots in Chengduo County (Yushu Prefecture), showed closer phylogenetic affinity to strain 245 Ade1 from Maqin County (Golog Prefecture) than to strain 217 Ade1 collected from the same Chengduo County location.

The analysis further revealed that *Adenoviridae* exhibits wide host distribution across humans, bats, dogs, and cattle. Notably, bat-associated *Adenoviridae* demonstrated closer phylogenetic proximity to human-associated strains compared to other host species, suggesting heightened zoonotic transmission risk from bat reservoirs. These findings highlight significant genetic diversity within *Adenoviridae* and indicate substantial evolutionary potential for host adaptation, which may critically influence viral transmission dynamics and zoonotic emergence risks.

### Phylogenetic analysis of *Astroviridae*

Members of the family *Astroviridae*, causative agents of gastroenteritis in humans and animals, possess a single-stranded positive-sense RNA genome with ORF1a/ORF1b encoding the RNA-dependent RNA polymerase (RdRp) that mediates viral RNA synthesis (30). The RdRp gene’s evolutionary conservation and low mutational frequency establish it as a robust molecular marker for phylogenetic reconstruction.

**Fig 6:**
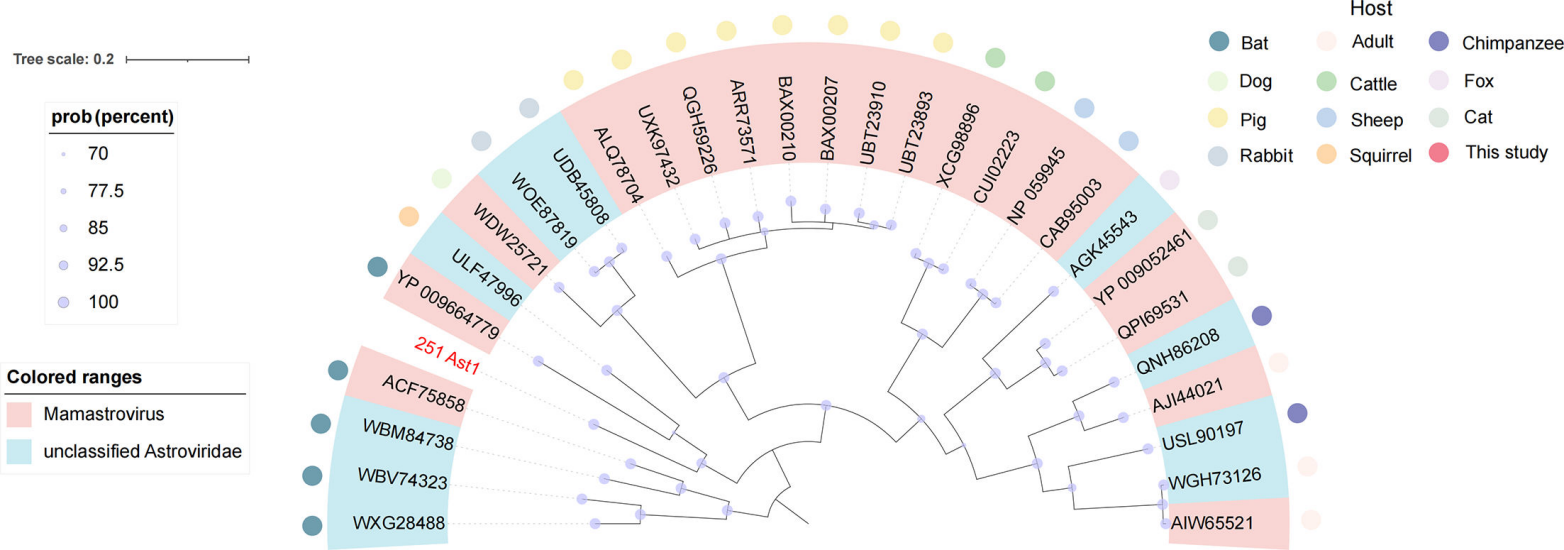
Phylogenetic reconstruction of *Astroviridae*. Evolutionary relationships were inferred using Bayesian methods based on RdRp amino acid sequences of *Astroviridae* members. Red branches denote sequences identified in the current investigation. The scale bar corresponds to amino acid substitutions per site.

The phylogenetic analysis (Fig. 6) revealed a near-complete *Astroviridae* genome (designated 251 Ast1) identified in this study, marked by a red node demonstrating clear evolutionary relationships with known astroviruses. This novel strain exhibits 72.76% sequence identity to ULF47996 from Chinese squirrels (2021) and 65.32% sequence identity to YP_009664779 from Guangxi bats (2007), highlighting *Astroviridae*’s broad mammalian host range. Given its low sequence similarity to established genera, 251 Ast1 has been provisionally classified as unclassified *Astroviridae*. Further discoveries of related strains may warrant the establishment of novel genera or subgenera, thereby expanding the *Astroviridae* taxonomic framework and enhancing our understanding of its evolutionary diversity.

### Phylogenetic analysis of *Parvoviridae*

**Fig 7:**
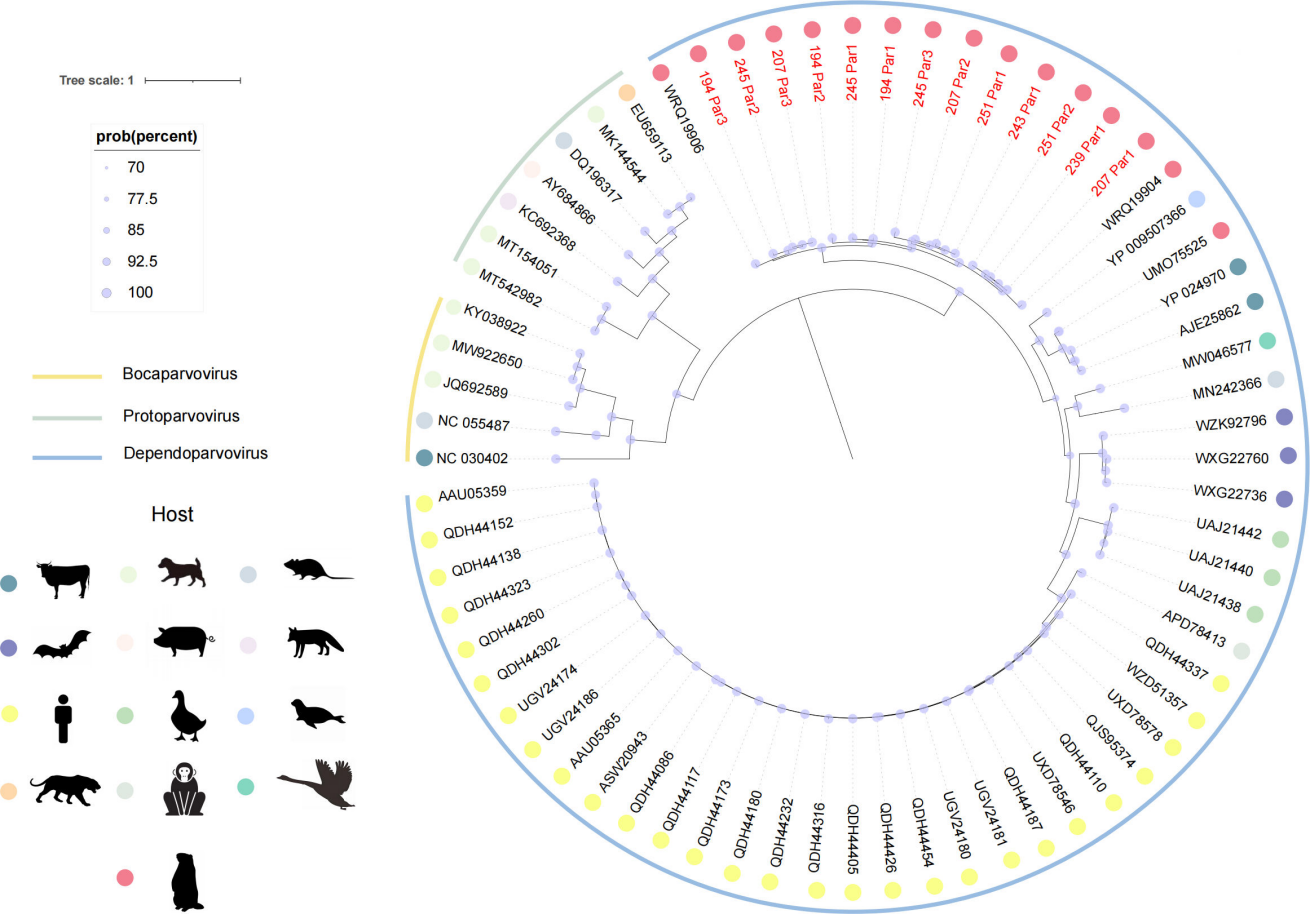
Phylogenetic relationships within *Parvoviridae*. Maximum likelihood tree generated from alignments of non-structural protein 1 (NS1) amino acid sequences. Red nodes indicate sequences characterized in this study. The scale bar represents amino acid substitutions per site.

The family *Parvoviridae* comprises small single-stranded DNA viruses in which the NS1 protein plays a central role in viral replication through its involvement in DNA synthesis and transcriptional regulation (31). The NS1 gene exhibits high evolutionary conservation and low mutation rates, making it a robust molecular marker for phylogenetic analysis to elucidate viral lineage relationships and evolutionary dynamics. This study identified 13 novel near-complete *Parvoviridae* genomes, with phylogenetic analysis (Fig. 7) demonstrating their evolutionary positions through red nodes that distinguish them from known viral lineages. The newly discovered strains clustered closely with WRQ19904 and WRQ19906, both identified in Xinjiang marmots (China, 2016), exhibiting >85% sequence identity. Notably, these 13 strains formed a distinct clade within the Dependoparvovirus genus, suggesting potential classification as a novel subgenus. Given Dependoparvovirus’s documented ability to infect humans, these findings underscore the genetic diversity within *Parvoviridae* and indicate ongoing viral evolution and host adaptation, which may significantly influence transmission dynamics and zoonotic risk potential (32).

### Phylogenetic analysis of *Picornaviridae*

**Fig 8:**
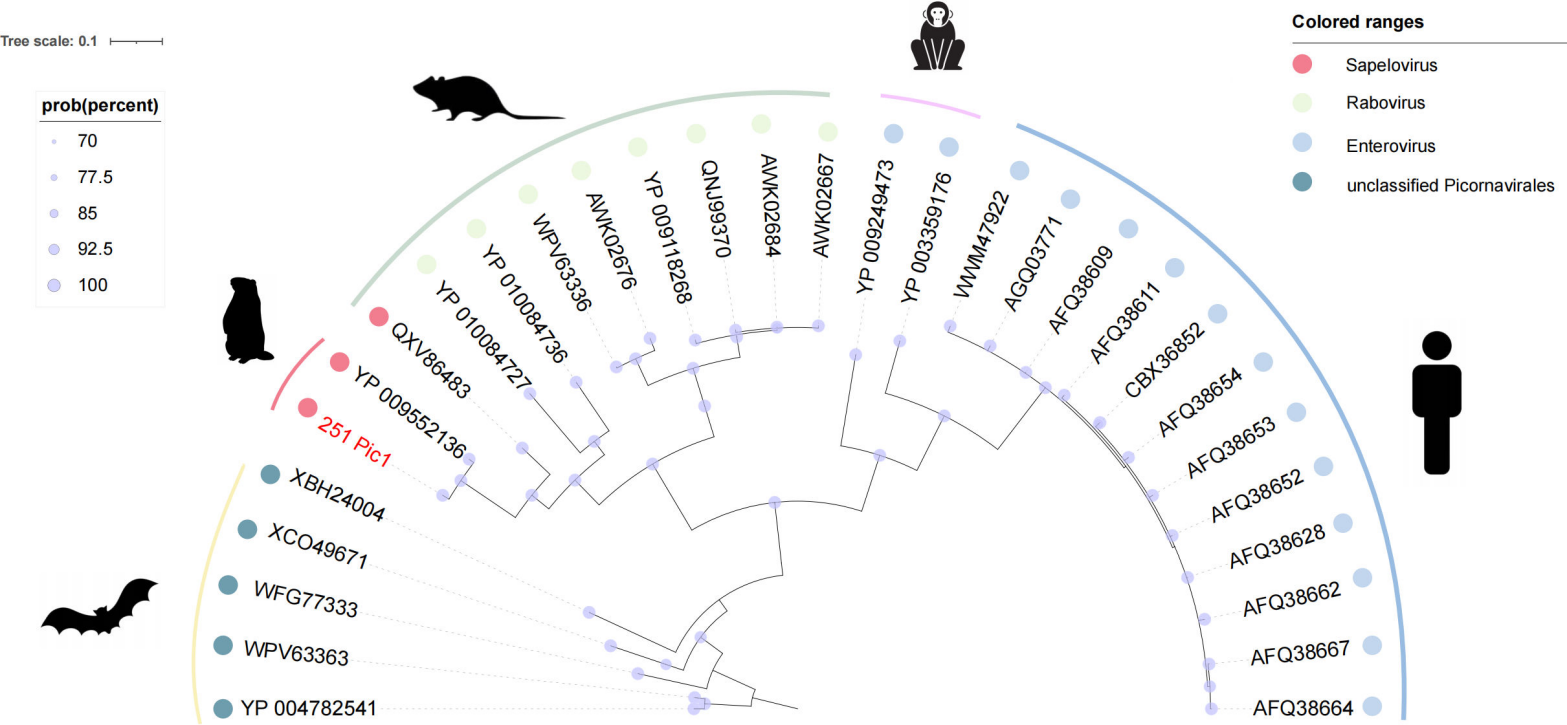
Phylogenetic analysis of *Picornaviridae*. Bayesian phylogenetic reconstruction based on RdRp amino acid sequence alignments, with sequences from this study marked in red. The scale bar shows amino acid substitutions per site, and colored branches correspond to different genera as indicated in the legend.

Members of *Picornaviridae* (single-stranded +RNA viruses) utilize conserved RdRp with low mutation rates, making this polymerase gene a key molecular marker for tracing viral evolution and transmission patterns across diverse hosts (33). This study identified a novel near-complete *Picornaviridae* genome (designated 251 Pic1), with phylogenetic analysis (Fig. 8) revealing its distinct phylogenetic positioning relative to known viral lineages (indicated by a red node). The new strain exhibits 97.78% sequence identity to YP_009552136, previously identified in Qinghai-Tibet Plateau marmots (China, 2013). Notably, 251 Pic1 clusters within the Sapelovirus genus, which predominantly circulates among marmots and rodents. While Sapelovirus transmission has not been documented in humans (unlike Enterovirus), the high mutability characteristic of RNA viruses underscores the importance of monitoring potential cross-species adaptation risks (34). This finding expands our understanding of *Picornaviridae* diversity and highlights the need for continued surveillance of viral evolution in animal reservoirs.

### Phylogenetic analysis of CRESS DNA viruses

**Fig 9:**
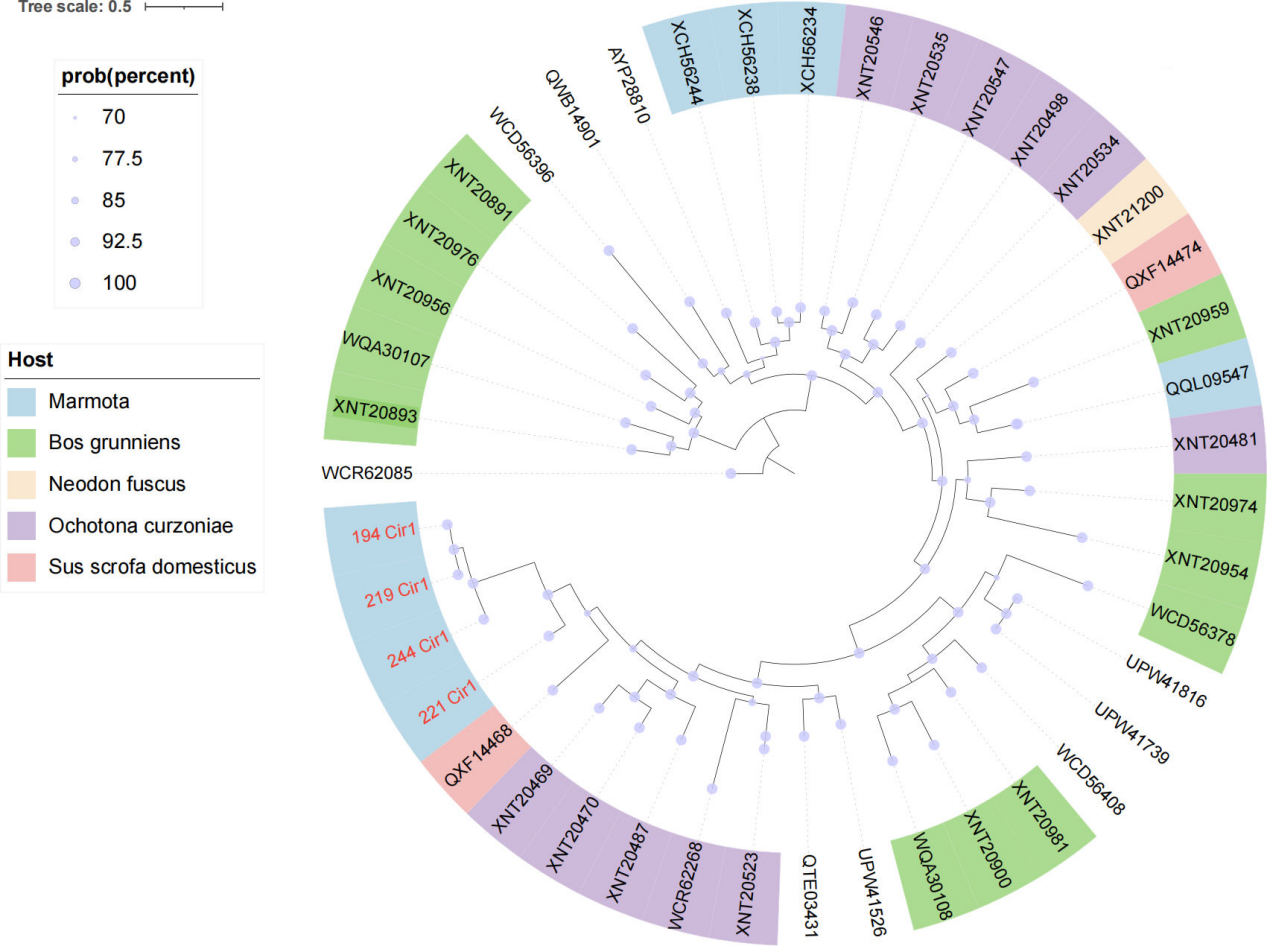
Evolutionary analysis of CRESS DNA viruses. Phylogenetic tree constructed using Rep protein amino acid sequences through Bayesian inference methods. Red nodes represent novel sequences identified in this work. The scale bar indicates amino acid substitutions per site.

CRESS DNA viruses utilize two core proteins: Rep (essential for rolling-circle replication) and Capsid (viral structure). The Rep gene’s high conservation and low mutation rate serve as a robust phylogenetic marker, revealing evolutionary relationships and replication mechanisms within this viral group (35). This study identified four novel complete CRESS DNA viral genomes. Phylogenetic analysis (Fig. 9) demonstrates their distinct evolutionary positions through red nodes differentiating them from known viral lineages. These newly discovered strains exhibit low sequence identity (<60%) to classified viruses within the unclassified Cressd group, forming a cohesive cluster that suggests potential classification as a novel genus. This finding expands the known diversity of mammalian-associated CRESS DNA viruses and provides foundational data for refining taxonomic frameworks within this viral group.

## DISCUSSION

The extraordinary genetic diversity and widespread ecological distribution of viruses establish them as fundamental yet undercharacterized components of ecosystems, particularly in extreme environments like high-altitude regions characterized by hypoxic conditions, extreme temperature variations, and intense UV radiation (4). These environments impose unique evolutionary pressures that shape viral communities through mechanisms requiring further elucidation. This investigation provides a comprehensive characterization of the gut virome in marmots inhabiting Qinghai Province’s high-altitude regions, yielding critical insights into viral diversity, community organization, and zoonotic emergence potential.

Analysis of 70 marmot gut samples revealed over 500 viral species, demonstrating exceptional virome diversity in high-altitude ecosystems. Dominant families included *Siphoviridae*, *Podoviridae*, *Myoviridae*, and *Microviridae*. The prevalence of *Siphoviridae* (temperate bacteriophages) across all samples highlights their role in gut homeostasis and viral community assembly. This pattern suggests that marmot guts harbor both pathogenic and symbiotic viruses that influence microbial dynamics and host immunity. Geographical stability of *Siphoviridae* distribution indicates ecological resilience in high-altitude environments, potentially reflecting phage-bacteria coevolution and microbiome adaptation to environmental stressors.

Geographical variations in viral family distributions were observed, with Chengduo County showing *Podoviridae* predominance and Maqin County exhibiting elevated *Myoviridae*/*Microviridae* levels. The significant enrichment of *Podoviridae* in the Chengduo group, as identified by LEfSe with the highest LDA score, suggests a pivotal role of this viral family in shaping the viral community structure between the Chengduo and Maqin groups. The strong association of *Podoviridae* with the Chengduo group could reflect environmental or host-specific factors that favor its proliferation or persistence. Moreover, the prominent contribution of *Podoviridae* to group differentiation underscores its potential as a biomarker for environmental or health-related variations within these populations. These geographical patterns emphasize the critical need to consider localized ecological variables—particularly altitudinal gradients, microclimatic conditions, and ecosystem characteristics—when analyzing wildlife viral community assembly mechanisms.

The α-diversity analysis showed no significant differences in species richness or evenness between the two regions, suggesting that the overall diversity of viral species in the marmot gut microbiome is relatively consistent across geographical locations. However, the significant β-diversity divergence between Chengduo and Maqin counties, as revealed by comparative analysis, highlights distinct ecological patterns in viral community structure. These differences are likely reflective of the unique environmental pressures that define each region. The observation that viral community composition varies substantially across regions with similar α-diversity indices emphasizes the dynamic nature of viral populations, where shifts in species composition may occur without changes in overall diversity.

Phylogenetic analysis of the *Adenoviridae* family revealed the presence of novel viral strains closely related to known adenoviruses in humans and animals, highlighting their potential for cross-species transmission. This finding is particularly significant given the association of adenoviruses with respiratory and gastrointestinal diseases in both human and animal hosts (36). Marmot-derived adenovirus strains exhibited close phylogenetic relationships to previously identified strains in other rodent populations, suggesting a broad host range across multiple mammalian species. The detection of these novel adenoviruses in marmots inhabiting high-altitude ecosystems raises concerns about zoonotic transmission risks, especially in regions where wildlife and human populations overlap. Notably, bat-derived adenoviruses displayed closer phylogenetic proximity to human strains than to those from other species, further emphasizing the zoonotic potential of these viruses and reinforcing the role of bats as critical viral reservoirs.

Similarly, analysis of the *Astroviridae* family identified a novel strain sharing significant sequence identity with astroviruses from squirrels and bats. As established causative agents of gastroenteritis in humans and animals, the detection of this strain in marmots expands current knowledge of astrovirus host diversity (37). The identification of this divergent strain in a high-altitude ecosystem suggests that these environments may harbor previously unrecognized viral lineages with zoonotic potential. The limited sequence similarity to known astroviruses implies rapid evolutionary dynamics within this viral family, which could drive the emergence of novel variants capable of infecting new host species.

The discovery of 13 novel *Parvoviridae* strains is particularly notable, given the documented zoonotic capacity of this viral family to infect humans, domestic animals, and wildlife (38). Phylogenetic clustering of these strains with marmot-associated parvoviruses from other regions of China indicates potential viral circulation within localized wildlife populations, highlighting their role as reservoirs for zoonotic spillover. The evolutionary conservation of the NS1 protein provides a robust molecular marker for tracing viral evolution and host adaptation mechanisms, which are critical for predicting emerging zoonotic variants. Enhanced surveillance of parvoviruses in marmot populations is warranted to assess their zoonotic risk.

A novel *Picornaviridae* strain closely related to Sapelovirus was identified, expanding current understanding of picornavirus diversity in high-altitude rodents. Although Sapelovirus has not been linked to human disease, its genetic plasticity and transmission potential at wildlife-livestock-human interfaces necessitate ongoing surveillance (33). The characteristic high mutation rate of picornaviruses facilitates rapid host adaptation, underscoring the need to monitor evolutionary trajectories that might enhance zoonotic capacity.

Finally, novel CRESS DNA viruses detected in marmot gut samples substantially broaden the known diversity of this understudied viral group. The low sequence identity (<60%) to characterized CRESS DNA viruses suggests that these strains may represent a novel genus or subgenus, emphasizing the need for taxonomic reclassification. High conservation of the Rep gene, combined with its low mutation rate, offers a reliable marker for evolutionary tracing and cross-species transmission risk assessment. These findings collectively underscore the importance of wildlife reservoirs in viral emergence and advocate for comprehensive surveillance systems integrating both characterized and unclassified viral taxa.

However, metagenomic analysis of gut viromes remains limited by incomplete viral genome assembly due to high viral diversity and complexity. Reference databases are biased toward known viruses, hindering accurate classification of novel or divergent strains. Additionally, metagenomics cannot distinguish active infections from latent or free viral particles, and methodological variations in sample processing and data analysis affect reproducibility. These challenges underscore the need for improved databases and standardized approaches to enhance gut virome research.

In conclusion, this study provides a comprehensive analysis of the gut virome of marmots inhabiting high-altitude regions of Qinghai Province, revealing novel viral strains and highlighting the intricate relationship between environmental pressures, host biology, and viral evolution. The findings underscore the importance of high-altitude ecosystems as reservoirs for diverse viral communities, including potential zoonotic pathogens. Given the interconnectedness of wildlife, livestock, and human populations in these regions, our results emphasize the need for integrated surveillance strategies to monitor viral diversity and predict potential zoonotic spillover events.

## Data Availability

The viral metagenomic data utilized to corroborate the findings of this study has been submitted and deposited at the National Genomics Data Center. The quality-filtered sequence reads are accessible in the Sequence Read Archive and can be found under the BioProject ID PRJCA035268 and the BioSample IDs SAMC4593282-SAMC4593351. All newly identified genes have been approved by the National Genomics Data Center and assigned sequence IDs. The serial numbers were C_AA105068–C_AA105090. It is important to note that there are no access restrictions imposed on these data.
